# Analytical and physiological validation of an enzyme immunoassay to measure oxytocin in dog, wolf, and human urine samples

**DOI:** 10.1038/s41598-021-92356-z

**Published:** 2021-06-17

**Authors:** G. Wirobski, F. S. Schaebs, F. Range, S. Marshall-Pescini, T. Deschner

**Affiliations:** 1 Domestication Lab, Wolf Science Center, Konrad-Lorenz-Institute for Ethology, University of Veterinary Medicine, Veterinaerplatz 1, 1210 Vienna, Austria; 2 Interim Group Primatology, Max-Planck-Institute for Evolutionary Anthropology, Deutscher Platz 6, 04103 Leipzig, Germany; 3 University of Leipzig, ZLS, Prager Str. 34, 04317 Leipzig, Germany

**Keywords:** Biochemistry, Biological techniques, Physiology, Biomarkers, Endocrinology

## Abstract

Oxytocin (OT) promotes pro-sociality, bonding, and cooperation in a variety of species. Measuring oxytocin metabolite (OTM) concentrations in urine or saliva provides intriguing opportunities to study human and animal behaviour with minimal disturbance. However, a thorough validation of analytical methods and an assessment of the physiological significance of these measures are essential. We conducted an analytical validation of a commercial Enzyme Immunoassay (EIA; Arbor OT assay kit) to measure OTM concentrations in dog, wolf, and human urine samples. To test the assay’s ability to detect changes in OTM concentrations, we administered oxytocin intranasally to 14 dogs. Assay performance with regard to parallelism was acceptable. Assay accuracy and extraction efficiency for dog and wolf samples were comparable to a previously validated assay (Enzo OT assay kit) but variation was smaller for human samples. Binding sensitivity and antibody specificity were better in the Arbor assay. Average OTM concentrations were more than twice as high as in comparable samples measured with the Enzo assay, highlighting a lack of comparability of absolute values between different assays. Changes in OTM concentrations after intranasal treatment were detected reliably. The Arbor assay met requirements of a “fit-for-purpose” validation with improvement of several parameters compared to the Enzo assay.

## Introduction

The measurement of peripheral oxytocin (OT) concentrations has become a widespread tool in psychology and animal behaviour research^1–3^. Previous work in humans has associated endogenous OT release with trust^4,5^, mother–child play^6^, and social affiliative touch^7^. Research with non-human animals has linked peripheral OT concentrations to prosocial and affiliative behaviour^8,9^, inter-specific interactions involving social touch^10,11^, and domestication^12^. OT is a neuropeptide hormone that regulates physiological processes such as eating behaviour and satiety^13^, heart rate and blood pressure^14^, birth, lactation, and parenting behaviour^15,16^, and also plays a crucial role in social bond formation and maintenance^17^. It is produced in the hypothalamus and released into the bloodstream by the pituitary gland, hence it can be measured centrally (in brain tissue by microdialysis^18^; in cerebrospinal fluid^19^) or in peripheral substrates (in plasma^20^; in milk^21^; in saliva^22^; in urine^9^). Non-invasive means of measuring OT concentrations (i.e., in saliva and urine) are in high demand because they do not disturb the subject’s behaviour and are less likely to cause a stress response which may in turn affect OT concentrations^23,24^.

However, the validity of studies measuring peripheral OT concentrations has been criticized^25–28^, with many published studies not reporting the essential validation steps of the assays used to measure OT and/or its metabolites, suggesting that inconsistent findings are likely associated with a lack of analytical rigor and consistency^26^. Comparability across studies and labs is severely hindered by the fact that there are no standardized protocols detailing how to prepare samples to measure OT and its metabolites in different sample matrices and/or species. For example, as demonstrated by a recent meta-analysis^29^, it is particularly important to state whether or not sample extraction has been conducted before analysis as this greatly affects measurements. There are several different ways to measure OT and its immunoreactive metabolites in peripheral substrates, including enzyme immunoassays (EIA^9^), radio immunoassays (RIA^30^), and mass spectrometry applications (i.e., LC–MS^31,32^; nanoLC-MS^33^). The current paper will focus on EIAs as they appear to be most commonly used in the behavioural sciences and psychology, yet to date only a few published studies conducted and reported validations for OT EIAs using peripheral substrates (Table 1).

**Table 1:** Overview of studies reporting validations of oxytocin EIAs using peripheral substrates (blood, urine, saliva).

Reference	Species	Substrate	Assay provider	Parameters reported
Péqueux et al.^46^	Human	Plasma	In-house	Parallelism, sensitivity, specificity, precision (CVs), IR
Kramer et al.^47^	Rat, vole	Plasma (not extracted)	Enzo*	Parallelism, accuracy, precision (CVs), physiological validation (injection of OT)
Snowdon et al.^8^	Tamarin	Urine	Enzo*	Parallelism, accuracy, precision (CVs), physiological and biological validation (estradiol pellets, social isolation)
Szeto et al.^48^	Human	Plasma	Enzo*	Comparison of extracted vs. unextracted samples using RIA and EIA: extraction efficiency, precision (CVs), assay accuracy/sensitivity, linearity, IR
Robinson et al.^23^	Seal	Plasma	Enzo*	Comparison of vacutainer types and extracted vs. unextracted samples; extraction efficiency, precision (CVs), physiological validation (injection of OT)
Reyes et al.^42^	Human	Urine	Enzo*	Effects of repeated freeze–thaw cycles, dehydration, acidity; dilution linearity, precision (CVs)
Bienboire-Frosini et al.^49^	Dog, cat, horse, pig, goat, sheep, cattle	Plasma	Enzo*	Extraction efficiency, sensitivity/quantification ranges, precision (CVs), dilution linearity (dog, cat)
Benítez et al.^50^	Capuchin monkey	Urine	Enzo*	Extraction efficiency, linearity, precision (CVs), physiological and biological validation (intranasal OT, grooming, fur-rubbing)
MacLean et al.^22^	Dog	Saliva	Arbor^+^ Cayman^#^ Enzo*	Parallelism, linearity, accuracy, precision (CVs), IR, comparison of extracted and unextracted samples, evaluation of sample collection techniques (swabs, saliva stimulation, food), biological validation and correlation between plasma and saliva OT (lactation/nursing)
Leeds et al.^51^	Gorilla	Saliva, urine	Arbor^+^	Parallelism, recovery, precision (CVs), comparison of extracted and unextracted samples, evaluation of diurnal variation, physiological and biological validation (intranasal OT, play, breeding, conspecific death)
Summarized in Ziegler^3^	Human, chimpanzee, baboon, tamarin, marmoset	Plasma, urine	Enzo*	Parallelism, accuracy, recovery
Schaebs et al.^36^	Wolf, dog	Urine	Enzo*	Evaluation of storage stability, repeated freeze–thaw cycles, addition of phosphoric acid, extraction protocol; parallelism, dilution linearity, accuracy, extraction efficiency, precision (CVs), repeatability, IR
Moscovice et al.^52^	Bonobo	Urine	Enzo*	Parallelism, dilution linearity, accuracy/recovery, precision (CVs)
Lürzel et al.^11^	Cattle, pig, goat	Saliva	Cayman^#^	Parallelism, accuracy/recovery, precision (CVs), comparison of extracted and unextracted samples; biological validation (positive human-animal interaction)
López-Arjona et al.^53^	Pig	Saliva	In-house	Accuracy (dilution linearity, recovery), precision (CVs), quantification of detection range, comparison of extracted and unextracted samples; biological validation (post farrowing/lactation)
Murata et al.^54^	Dog	Serum, urine	In-house	Antibody affinity, dilution linearity, recovery, quantification of detection range
Schaebs et al.^37^	Human	Urine	Enzo*	Evaluation of storage stability, repeated freeze–thaw cycles, addition of phosphoric acid; parallelism, accuracy, extraction efficiency, precision (CVs), IR

*Enzo Life Sciences, Assay Designs Inc., Ann Arbor, MI, USA, https://www.enzolifesciences.com, ^+^ Arbor Assays Headquarters, Ann Arbor, MI, USA, https://www.arborassays.com, ^#^ Cayman Chemical, Ann Arbor, MI, USA, https://www.caymanchem.com.

In general, one can differentiate between full and partial validations: A full validation is necessary when establishing a new assay for the first time, or when a commercially available assay kit is used for the first time for a particular species and/or sample matrix. A partial validation may be sufficient when a commercial assay is used and the manufacturer has already assessed certain parameters (such robustness or antibody cross-reactivity) during development^34^. Nevertheless, each assay needs to be validated every time, prior to its use, in a different species, for each new sample matrix, or when a new extraction protocol is established. The following is usually needed to sufficiently validate an immunoassay for its intended use: an assessment of its (1) selectivity (i.e., antibody cross-reactivity), (2) dilution linearity or parallelism (i.e., to determine the assay’s linear range by using either spiked or non-spiked samples, and identify potentially interfering matrix effects, respectively), (3) extraction efficiency and assay accuracy to calculate percent recovery and variation, (4) performance of a biological or physiological validation using a known trigger of endogenous OT release or by administering exogenous OT, and finally, (5) assessment of antibody specificity using chromatographic separation^3^. Furthermore, recording measures of repeatability and precision (i.e., reported as intra- and inter-assay coefficients of variation (CV) and on-going internal quality control (QC) are required for continuous evaluation of assay performance throughout a study including publication of obtained values alongside results. A validation should reflect the intended purpose of a subsequent study and may be considered successful if it produces reliable results in the context of the data’s intended use (see ‘fit-for-purpose approach’ in biomarker research^34,35^). It should also allow the estimation of the smallest detectable effect to determine whether the assay is suitable given the expected effect size of a study. Lastly, even if an assay does not meet requirements for a given purpose, validation parameters should be reported nonetheless, as this information may contribute to saving valuable resources.

The aim of the present paper was to analytically and physiologically validate a commercially available OT EIA kit (Arbor Assays, Ann Arbor, MI, USA, Cat. No. K048-H5) for dog, wolf, and human urine samples, and compare its performance to another commercial kit (Enzo Life Sciences, Assay Designs, Cat. No. 901-153A-0001) previously validated for OTM measurement in dog and wolf^36^ as well as human urine^37^ by our group, thereby providing practical recommendations for future studies. To this end, we ran tests of parallelism for each species to investigate the presence of matrix effects. Next, we assessed extraction efficiency and assay accuracy followed by the determination of patterns of immunoreactivity (IR). Finally, we physiologically validated the assay by intranasally administering exogenous OT (or a placebo) to a group of pet dogs. All analytical parameters for the Enzo assay kit reported in this paper were obtained in the same way as for the Arbor assay. We used pooled samples from the same study populations for analytical validation of both assays; however, we did not reuse the old samples from the Enzo validation to avoid long storage periods. All tests were conducted by the same experimenter under the same laboratory conditions. Full methodological details and results for the Enzo assay were published before^36,37^ and are cited here for comparative purposes.

## Material and methods

### Subjects

Urine samples of 11 pet dogs (5 females, 6 males) and 8 humans (4 females, 4 males) were collected at the Max-Planck-Institute for Evolutionary Anthropology (MPI EVA) in Leipzig, Germany, and urine samples of 6 wolves (3 females, 3 males) were collected at the Wolf Science Center (WSC), in Ernstbrunn, Austria, for analytical assay validation. All individuals were in good health status at the time of sample collection. For the physiological validation, 14 adult, healthy pet dogs of different breeds (9 males, 5 females) recruited from the database of the Clever Dog Lab (CDL) of the University of Veterinary Medicine (Vienna, Austria) were trained to inhale OT nasal spray (Syntocinon, Novartis) using a vaporizer mask previously shown to be effective in administering exogenous OT to dogs^38^.

### Urine sample collection

Dog urine samples at the MPI EVA were collected when the dogs urinated spontaneously during leashed walks with their owners in an outside area in front of the institute. Urine samples were collected in plastic trays (Carl Roth, 5195.1) and brought to the Endocrinology Laboratory within 5 min. Human participants were asked to urinate into a plastic tray (Carl Roth, 5195.1) and samples were then brought to the Endocrinology Laboratory at the MPI EVA, as well within 5 min following collection.

**Figure 1:**
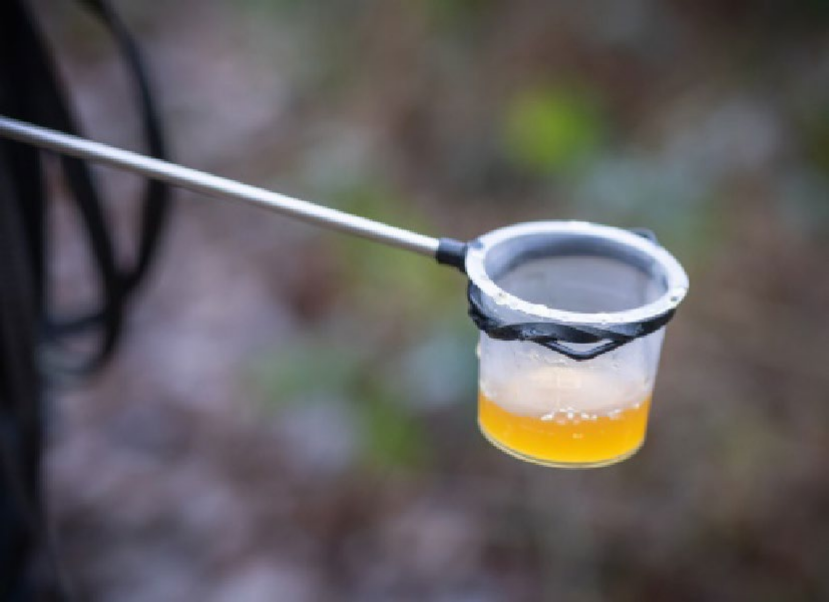
Urine collection device used for dogs and wolves consisting of an expandable metal stick and a plastic cup.

Dogs at the CDL and wolves at the WSC (once habituated to the urine collection process using an expandable metal stick with a plastic cup attached; Carl Roth, 5195.1; Fig. 1) provided spontaneously voided urine samples during leashed walks with their owners or animal trainers, respectively. Within a maximum of 15 min following collection, samples (kept on ice packs in the meantime) were brought to the facilities of the CDL or WSC.

At the respective laboratories, all urine samples (dog, wolf, and human) were subsequently divided into 1 ml aliquots and 100 µl of a 0.1% phosphoric acid (PA) was added per 1 ml sample to avoid OT degradation^36,37^. Samples were aliquoted and frozen at − 20 °C until further processing. In case samples had to be transported to the MPI EVA for extraction and analysis, they were kept on dry ice during shipment which took less than 12 h.

### Intranasal oxytocin administration

To physiologically validate the assay at hand, we administered 12 international units (IU) OT nasal spray (Syntocinon, Novartis) or a placebo (PL; saline solution; 0.9% sodium chloride, Ringer) using a vaporizer mask (Nebutec, M-neb vet nebulizer and inhalation mask for dogs; see Schaebs et al.^38^ for details) to 14 pet dogs and collected urine samples before and 45–60 min after treatment. Each dog received both treatments in a semi-randomized and counterbalanced order, on different days. Analysis of the samples was blinded (i.e., the experimenter processing the samples did not know which treatment the dog had received).

### Ethics declarations

#### Wolves and dogs

The study was discussed and approved by the institutional ethics and animal welfare committee and all experiments were performed in compliance with GSP and ARRIVE guidelines and national legislation. Specifically, approval was obtained from the ethical commission of the University of Veterinary Medicine, Vienna (approval number: ETK 05/03/2017) for the wolf samples, and from the ethical commission of the Max Planck Society for the dog samples (approval number 2017_07) used for the analytical assay validation. The OT/PL administration was part of a study with pet dogs run at the CDL (University of Veterinary Medicine, Vienna) and approved by its ethical commission (approval number: ETK 13/11/2017). We obtained informed consent from all pet dog owners after full description of the procedure.

#### Human participants

The study was discussed and approved by the institutional ethics committee and all experiments were performed in accordance with GSP guidelines and national legislation. Ethical approval for participation of human subjects was obtained from the ethical commission of the Max Planck Society (approval number 2017_09) and informed consent was obtained from all participants after full explanation of the purpose and nature of the study.

### Sample extraction and urinary oxytocin metabolite measurement

All laboratory analyses were performed in the Endocrinology lab at the MPI EVA. Urine sample extraction with solid phase extraction (SPE) cartridges was conducted according to a previously validated and published protocol^9^ incorporating minor adjustments (see^36^ for details). Extracted samples were analysed according to the assay manufacturer’s instructions and incubated overnight at 4 °C. All samples were measured in duplicates. When optical density (OD) values of sample duplicates differed more than 10% the measurement was repeated or the sample got excluded from further analysis.

Average Zero standard (B0; wells contained only assay buffer but no sample) OD values achieved after incubation were more than twice as high with the Arbor as with the Enzo assay1.11 (SD 0.12; N = 12 plates) for the Arbor, and 0.47 (SD 0.08; N = 32 plates) for the Enzo assay, respectively.

The inter-assay CV of OTM concentrations for a high concentrated OT standard (QC high: 640 pg/ml; N = 5 plates) was 4.1%, and 16.8% for a low concentrated OT standard (QC low: 102.4 pg/ml; N = 5 plates). The intra-assay CV, as calculated by averaging variability across duplicates of all samples measured on a single assay plate, was 8.6% (N = 29 samples) for dog and wolf samples, and 9.5% (N = 29 samples) for human samples.

### Analytical validation

#### Parallelism

We conducted a test for parallelism for each of the three species to investigate the potential presence of matrix effects. 450 µl of an extracted dog urine pool was spiked with 50 µl of an OT standard (concentration 1600 pg/ml; supplied by Arbor Assays) and diluted serially^36^. The same procedure was performed on an extracted wolf and human urine pool.

#### Extraction efficiency and assay accuracy

To determine extraction efficiency and assay accuracy, we created five pools of dog, wolf, and human urine samples. For extraction efficiency, 237.5 µl pooled urine samples were spiked with 12.5 µl of three different concentrations of an OT standard (delivered with the assay system; high: 40,000 pg/ml; medium: 20,000 pg/ml; low: 10,000 pg/ml) before extraction. To assess assay accuracy, 237.5 µl extracted urine samples were spiked with 12.5 µl of the same three different concentrations of an OT standard (see above). Subsequently, percent recovery was calculated following the formula reported in^36^.

#### Immunograms

Patterns of immunoreactivity (IR) were investigated following the protocol given in^36^. In brief, IR was determined by running 100 µl of extracted dog, wolf, human pool samples, or extracted OT standard, over a Waters Alliance 2695 high-performance liquid chromatograph (HPLC) equipped with a Gemini C18 column (Phenomenex, Torrance, CA, USA). The obtained fractions were collected with a Waters Fraction Collector 3 (Waters, Milford, MA, USA), lyophilized overnight, and kept frozen at − 20 °C until measurement with the EIA. We calculated the percentage of ‘explained IR’ (i.e., IR that overlapped with the OT standard and thus likely originates from OT or one of its degradation products/metabolites) according to the formula given in^36^.

### Statistics

All statistical tests were run and plots created using R (version 3.3.3; paired t-tests performed using version 4.0.2^39^). We tested for parallelism by fitting a linear model including the interaction between sample type (standard curve and pooled sample) and the concentration of the standard with the percent binding as response variable^36^. The model was fitted using the function *lm*. The check for assumptions of normality and homogeneity of the residuals did not indicate any problems (inspection of a qq-plot of the residuals and residuals plotted against fitted values^40^). Model stability was assessed by means of DFBeta^40^, which did not indicate any problems. Paired t-tests were conducted to assess changes in urinary OTM concentrations from pre- to post-treatment using the data obtained from the physiological validation (intranasal OT administration). Effect sizes were determined using R^2^ (paired t squared/(paired t squared + df)).

## Results

The Arbor assay measured average OTM concentrations of 398 pg/ml (SD 158) in the dog urine pool, 367 pg/ml (SD 189) in the wolf urine pool, and 119 pg/ml (SD 62) in the human urine pool. In contrast, the Enzo assay measured average OTM concentrations of 152 pg/ml (SD 67) in pooled dog urine, 123 pg/ml (SD 47) in pooled wolf urine, and 35 pg/ml (SD 8) in pooled human urine^36,37^.

### Parallelism

**Figure 2:**
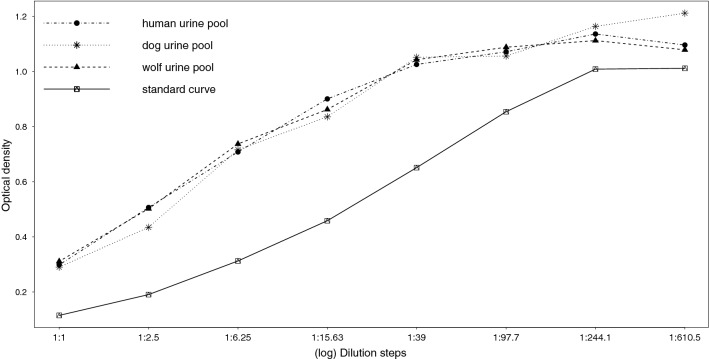
Parallelism of serially diluted human, dog, and wolf urine pool samples to the oxytocin (OT) standard curve. Note that the x-axis is on a log scale.

All three serially diluted pools were parallel to the standard curve (dog urine: t(12) =  − 0.233, P = 0.820; wolf urine: t(12) =  − 0.243, P = 0.812; human urine: t(12) =  − 0.351, P = 0.732) and this was confirmed by visual inspection (Fig. 2).

### Extraction efficiency and assay accuracy

For the dog urine pool, mean extraction efficiency was 138% (range: 127–144%; SD = 9.9; n = 3; Table 2) when spiked with a high, 137% (range: 115–157%; SD = 21.3; n = 3; Table 2) when spiked with a medium and 157% (range: 130–170%; SD = 22.7; n = 3; Table 2) when spiked with a low concentrated OT standard. Mean assay accuracy for the dog pool was 166% (range: 150–186%; SD = 18.0; n = 3; Table 2) when spiked with a high, 137% (range: 126–147%; SD = 15.1; n = 2; Table 2) when spiked with a medium and 137% (range: 134–140%; SD = 4.1; n = 2; Table 2) when spiked with a low concentrated OT standard.

**Table 2:** Extraction efficiency and assay accuracy for the Arbor and Enzo assays.

ARBOR extraction efficiency	Spiked concentration*	Dog	Wolf	Human
	Low	157% (range: 130–170%; SD = 22.7; n = 3)	119% (range: 96.8–152%; SD = 21.5; n = 5)	98.6% (range: 93.0–104%; SD = 8.0; n = 2)
	Medium	137% (range: 115–157%; SD = 21.3; n = 3)	132% (range: 118–143%; SD = 10.6; n = 4)	99.6% (range: 97.2–102%; SD = 2.6; n = 3)
	High	138% (range: 127–144%; SD = 9.9; n = 3)	119% (range: 111–128%; SD = 6.3; n = 5)	105% (range: 102–107%; SD = 2.5; n = 3)

**ARBOR assay accuracy**	Spiked concentration*	**Dog**	**Wolf**	**Human**
	Low	137% (range: 134–140%; SD = 4.1; n = 2)	143% (range: 123–162%; SD = 19.5; n = 3)	114% (range: 101–130%; SD = 14.7; n = 3)
	Medium	137% (range: 126–147%; SD = 15.1; n = 2)	132% (range: 119–149%; SD = 13.6; n = 5)	116% (range: 111–126%; SD = 8.6; n = 3)
	High	166% (range: 150–186%; SD = 18.0; n = 3)	129% (range: 115–146%; SD = 15.3; n = 5)	112% (range: 110–113%; SD = 1.7; n = 3)

**ENZO extraction efficiency**	Spiked concentration*	**Dog** ^36^	**Wolf** ^36^	**Human** ^37^
	Low	125% (range: 67.7–197%, SD = 46.9, n = 5)	109% (range: 62.9–140%, SD = 37.6, n = 4)	101% (range: − 16.2–156%, SD = 67.8, n = 5)
	Medium	157% (range: 137–175%, SD = 14.2, n = 5)	137% (range: 116–157%, SD = 20.8, n = 3)	98.9% (range: 66.9–131%, SD = 23.2, n = 5)
	High	132% (range: 121–154%, SD = 15.2, n = 4)	132% (range: 124 –145%, SD = 10.9, n = 3)	92.8% (range: 71.9–120%, SD = 19.4, n = 5)

**ENZO assay accuracy**	Spiked concentration*	**Dog** ^36^	**Wolf** ^36^	**Human** ^37^
	Low	156% (range: 106–246%, SD = 57.7, n = 5)	144% (range: 66–185%, SD = 54.2, n = 4)	113% (range: 74.8–190%, SD = 47.0, n = 5)
	Medium	164% (range: 144–173%, SD = 12.1, n = 5)	114% (range: 83–163%, SD = 42.9, n = 3)	120% (range: 104–140%, SD = 14.8, n = 5)
	High	145% (range: 129–170%, SD = 16.3, n = 5)	98.5% (range: 68.7–117%, SD = 26.1, n = 3)	126% (range: 108–160%, SD = 21.4, n = 5)

*237.5 µl pooled urine samples spiked with 12.5 µl of differently concentrated OT standard (high: 40,000 pg/ml; medium: 20,000 pg/ml; low: 10,000 pg/ml).

For the wolf urine pool, mean extraction efficiency was 119% (range: 111–128%; SD = 6.3; n = 5; Table 2) when spiked with a high, 132% (range: 118–143%; SD = 10.6; n = 4; Table 2) when spiked with a medium and 119% (range: 96.8–152%; SD = 21.5; n = 5; Table 2) when spiked with a low concentrated OT standard. Mean assay accuracy for the wolf pool was 129% (range: 115–146%; SD = 15.3; n = 5; Table 2) when spiked with a high, 132% (range: 119–149%; SD = 13.6; n = 5; Table 2) when spiked with a medium and 143% (range: 123–162%; SD = 19.5; n = 3; Table 2) when spiked with a low concentrated OT standard.

For the human urine pool, mean extraction efficiency was 105% (range: 102–107%; SD = 2.5; n = 3; Table 2) when spiked with a high, 99.6% (range: 97.2–102%; SD = 2.6; n = 3; Table 2) when spiked with a medium and 98.6% (range: 93.0–104%; SD = 8.0; n = 2; Table 2) when spiked with a low concentrated OT standard. Mean assay accuracy for the human pool was 112% (range: 110–113%; SD = 1.7; n = 3; Table 2) when spiked with a high, 116% (range: 111–126%; SD = 8.6; n = 3; Table 2) when spiked with a medium and 114% (range: 101–130%; SD = 14.7; n = 3; Table 2) when spiked with a low concentrated OT standard.

### Immunograms

**Figure 3:**
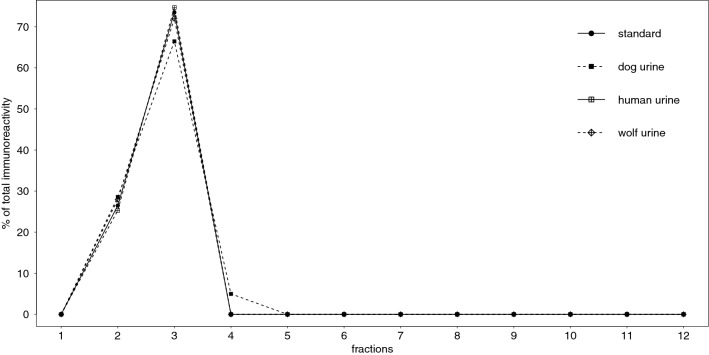
Percent of total immunoreactivity detected in each fraction in extracted oxytocin (OT) standard and extracted dog, wolf, and human urine.

The immunogram of the extracted OT standard revealed IR in fractions 2 and 3 (accounting for 26.5% and 73.5% of the total IR, respectively; Fig. 3, Table 3).

**Table 3:** Percent of total immunoreactivity (IR) detected in extracted urine explained by IR found in extracted oxytocin (OT) standard as measured by Enzo and Arbor OT assays.

**IR explained in %**	Enzo* ^36,37^ (%)	**Arbor**^**+**^** (%)**
Dog	80	95
Wolf	78	100
Human	98	100

*Enzo Life Sciences, Assay Designs Inc., Ann Arbor, MI, USA, https://www.enzolifesciences.com; ^+^Arbor Assays Headquarters, Ann Arbor, MI, USA, https://www.arborassays.com.

The immunogram of extracted dog urine revealed IR in fractions 2, 3 and 4 (accounting for 28.5%, 66.5% and 5% of the total IR, respectively; Fig. 3, Table 3). Thus, 95% of IR in extracted dog urine can be explained by that in extracted OT standard.

The immunogram of extracted wolf urine revealed IR in fractions 2 and 3 (accounting for 28% and 72% of the total IR, respectively; Fig. 3, Table 3). Thus, 100% of the IR found in extracted wolf urine can be explained by that in extracted OT standard.

The immunogram of extracted human urine revealed IR in fractions 2 and 3 (accounting for 25% and 75% of the total IR, respectively; Fig. 3, Table 3). Thus, 100% of the IR in extracted human urine can be explained by that in extracted OT standard.

### Physiological assay validation

**Figure 4:**
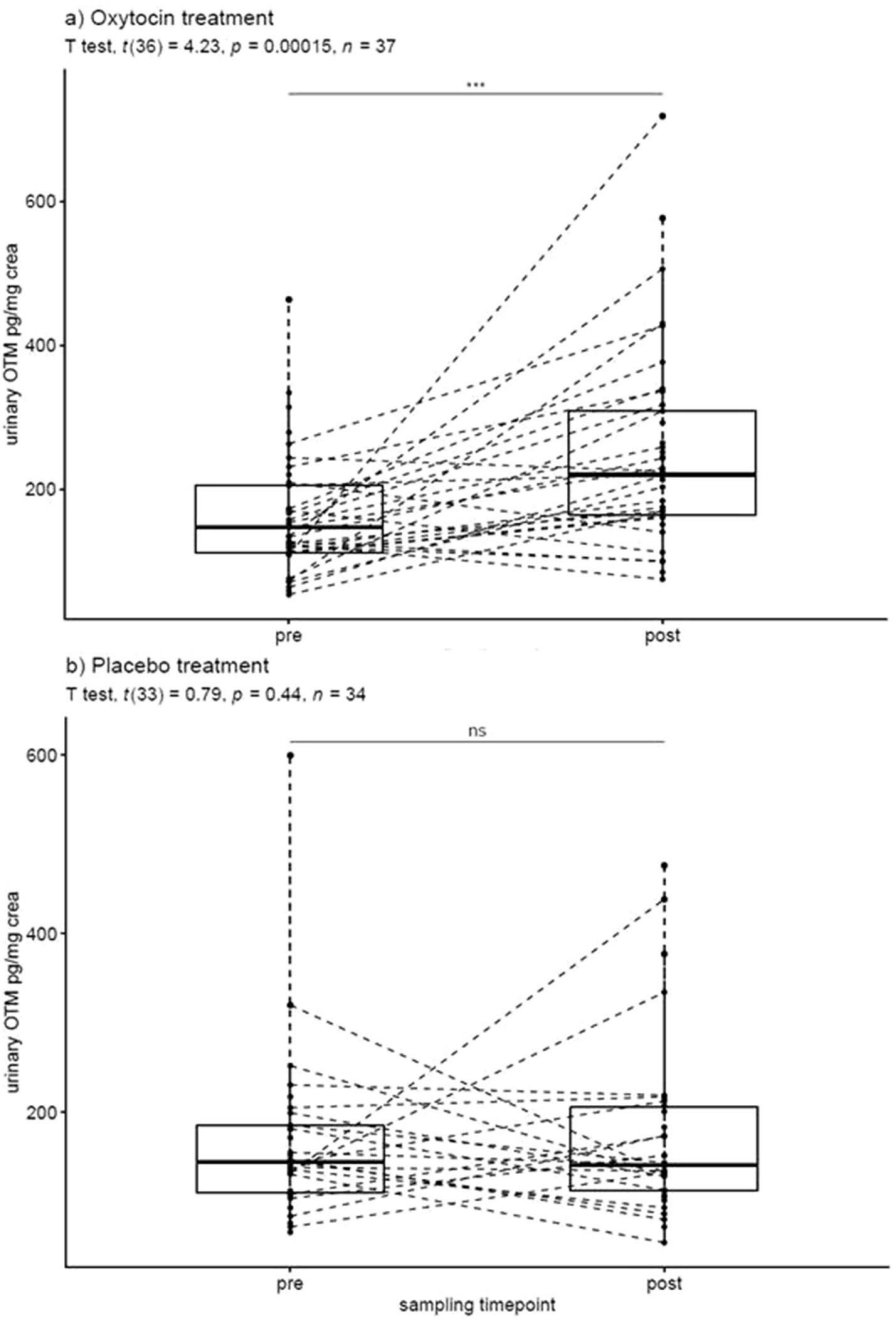
(**a**, **b**) Individual changes of urinary OTM concentrations following intranasal OT (**a**) or placebo (**b**) treatment in pet dogs (boxes indicate the interquartile range, horizontal lines indicate the median).

Following intranasal OT treatment, the Arbor assay measured an average increase in urinary OTM concentrations of 52.9% (average pre-treatment concentrations: 165 pg/mg creatinine; SD 86.1; range: 53.9–463; median = 147; average post-treatment concentrations: 252 pg/mg creatinine; SD 139; range: 75.2–718; median = 220). This increase was significant (t (36) = 4.23, P < 0.001) (Fig. 4a) and the effect size was large (R^2^ = 0.33). Following intranasal PL treatment, an increase in OTM concentrations of 4.9% was detected (average pre-treatment concentrations: 165 pg/mg creatinine; SD 94.3; range: 65.7–599; median = 144; average post-treatment concentrations: 173 pg/mg creatinine; SD 98.7; range: 54.1–476; median = 141). This increase was not significant (t (33) = 0.79, P = 0.44) (Fig. 4b) and the effect size was negligible (R^2^ = 0.02).

## Discussion

With the present study, we aimed to evaluate the performance of a commercial EIA kit (Arbor Assays, Ann Arbor) to measure urinary OTM concentrations in dogs, wolves, and humans. In addition, we tested whether the assay would pick up changes in dogs’ urinary OTM concentrations following intranasal treatment with either OT or a PL solution. Building on previous studies by our group^36,37^ we compare and discuss the outcomes of this validation of the Arbor OT assay in relation to the results we obtained for another commercial kit from a different manufacturer, the Enzo OT assay kit (Enzo Life Sciences, Assay Designs), to guide decisions regarding assay suitability for the measurement of urinary OTM in dogs, wolves, and humans.

The Arbor assay performed well with regard to inter- and intra-assay CVs and parallelism, in all three species assessed, indicating that matrix effects were not an issue. However, similarly to the Enzo OT assay, values for extraction efficiency and assay accuracy were higher than 100% for dogs and wolves for low, medium, and high concentrations with relatively large SDs (Table 2). For the human samples, values for extraction efficiency exceeded 100% only for the high concentration, but all three concentrations (low, medium, high) for assay accuracy. However, SDs for human samples were considerably lower compared to the Enzo assay. Taken together, results indicate a comparable performance of the two assays with regard to accuracy and extraction efficiency for dogs and wolves, but warrants caution when measuring samples in the lower range of both assays as subtle differences may not be picked up^36^. For human urine samples, the Arbor assay performed better than the Enzo with regard to its accuracy.

There was a striking difference in average Zero standard (B0) OD values achieved following over-night incubation. Compared to the Enzo assay, the Arbor assay reached OD values more than twice as high. Low OD readings due to insufficient colour development can be caused, among other things, by low temperature (in the lab, or of the reagents), too short incubation periods, or too many wash cycles, and may result in low repeatability (i.e., higher intra-assay CVs) as the standard curve becomes relatively flat and small differences in OD values result in largely different hormone concentrations. Furthermore, the proportion of measurements which fall below or above the linear range of the standard curve increases. This results in more samples needing to be re-measured. Therefore, for this aspect of binding sensitivity, the present assay showed clear advantages over the previously validated one.

To evaluate whether the assay system indeed measures OT and its immunoreactive metabolites rather than cross-reacting substances that do not stem from the OT metabolism, patterns of IR in the samples were determined. For the Arbor assay, the immunogram of OT standard showed one major peak in fraction 3 accounting for 73.5% of total IR, as well as considerable IR in fraction 2 accounting for 26.5% of total IR. OT molecules are sensitive to structural changes due to temperature and pH-level of the samples^41,42^ and may be altered or broken down during sample handling and extraction^3^. The finding of IR in more than one fraction of OT standard hence suggests the presence of not only OT, but also OT degradation products^36^. The immunograms for wolf and human urine revealed that IR was present in the same two fractions (fractions 2 and 3) as in the OT standard sample, explaining 28% and 72%, and 25% and 75% of total IR, respectively. In case of dog urine, IR was found in three fractions (2, 3, and 4), accounting for 28.5%, 66.5%, and 5% of total IR, respectively. Thus, while for wolf and human urine, 100% of IR in the samples can be explained by IR in extracted OT standard, for dog urine, only 95% of IR detected matched IR present in extracted OT standard and a small proportion of additional IR was found in fraction 4, accounting for 5% of total IR. Since all urine samples for the analytical assay validation were collected and treated exactly the same way from storage and extraction to measurement, this may reflect species-specific differences in either the metabolic breakdown of the OT molecule in the body, degradation processes during handling, particular features of the urine (i.e., such as acidity/pH-level^42^), or the presence of cross-reacting substances in dog urine that do not stem from OT metabolism. To investigate in detail how OT is metabolized in each species and secreted into specific substrates, one would have to perform a radiometabolism study whereby a radioactively labelled hormone is injected into an animal and samples are taken repeatedly to investigate excretion patterns (see for example^43^). Unfortunately such studies, while of great interest and importance, are often not feasible in the species at hand due to high invasiveness, budget considerations, and specific requirements related to handling radioactive material.

To summarize, proportions of IR in urine explained by IR patterns in the OT standard were considerably higher when samples were measured with the Arbor than the Enzo assay (Fig. 3, Table 3), in particular for wolf and dog samples, indicating higher antibody specificity and capacity to detect urinary OT and its metabolites/degradation products. This further suggests that the OT antibodies provided by the different manufacturers varied in the epitopes they recognized, hence different OT metabolites were detected by the two assays (see also^44^ for a comparison of two EIAs and a RIA), and emphasizes the lack of comparability of absolute hormones values across studies when different assay systems are used even if both assays were validated for the species and substrates at hand^45^. To illustrate this discrepancy, we found average OTM concentrations in the population pools (N = 11 dogs; N = 6 wolves; N = 8 humans) to be more than twice as high when comparable pool samples were measured with the Arbor than with Enzo assay^36,37^.

The Arbor assay was able to detect changes in pet dogs’ urinary OTM concentrations after intranasal treatment with OT nasal spray using a vaporizer mask and performed similarly to the previously validated assay^38^. Specifically, urinary OTM concentrations increased significantly following intranasal OT administration but not when a PL treatment was applied. Similar results were obtained with the Enzo assay^36^ and thus both assays appear suitable to determine administration success in studies using intranasal OT administration in dogs.

In addition to reporting assay validation parameters, Schaebs and colleagues^36^ outlined important factors to consider concerning sample storage (particularly regarding temperature and storage time) and highlighted the importance of sample extraction. Here we added the validation of another commercially available assay and found that both assays met the requirements of a “fit-for-purpose” validation^35^ and may be used to measure urinary OTM in dogs, wolves, and humans in behavioural or psychological research. The Arbor assay performed better with regard to binding sensitivity (i.e., maximum OD values achieved) and antibody specificity (proportions of IR explained). Hence, while further refinement of extraction protocols is still required to improve measures of accuracy, the assay system validated here may offer improved performance compared to the Enzo assay for the measurement of urinary OTM in dogs, wolves, and humans. Importantly, careful consideration of reported variation in assay accuracy and extraction efficiency in combination with CVs of QCs will allow estimation whether the assay system is accurate enough for a given study purpose particularly when expected effect sizes are known. To conclude, the present study further cautions against comparing absolute values across studies/labs when different assay systems were used and highlights the need for rigorous method validation in peripheral OT research before carrying out studies.

## Data Availability

The datasets generated during and/or analysed during the current study are available from the corresponding author on reasonable request.
